# A Problem in Eugenics

**Published:** 1905-10-28

**Authors:** 


### A Problem in Eugenics.

The hundredth anniversary of the death of the great Admiral who raised the naval glory of England to its highest point, and who died, not unfitly, at the culminating moment of his renown, is an event of so much interest and importance that even the medical journalist is compelled to turn aside, for the moment, from the topics which more immediately concern him, and to lay his tribute of laurel upon the tomb of the national hero, to whose merits time has done even more ample justice than was done by his contemporaries.

It is worth while to ask ourselves whence came Nelson, and whether his origin gives us any reason to hope that, in emergencies similar to those which he was called upon to encounter, the nation would find successors worthy to follow in his footsteps. His paternal descent was through a line of country squires and clergymen, who bore recorded arms from the fourteenth century, and who, in any Continental country, would have been considered and classed as "noble," but who, apart from this, do not appear to have been distinguished. His mother was the daughter of a reverend Prebendary of Westminster, Dr. Suckling, and her mother was a sister of the Earl of Orford, who is better known as Sir Robert Walpole, of Horatio, first Baron Walpole of Wolferton, and of a distinguished Admiral Walpole. One of Dr. Suckling's sons was also an admiral, who had done good service in his time, and it is said that Nelson's desire to enter the naval service was largely due to the example and the precept of this uncle. The Walpole pedigree is perhaps the most ancient and the most stainless in English records, and is carried back through a daughter of Alfred the Great to Charlemagne; but the Walpoles since the Conquest, with few exceptions, have been local magnates who have made no figure in history. Ralph de Walpole, in the thirteenth century, was Bishop of Norwich and afterwards of Ely; but the head of the family, for many successive generations, was no more than member of Parliament for the county of Norfolk or for one of its boroughs, and the husband of some lady of good descent in the locality. One of these ladies was Lucy, daughter of Sir Terry Robsart, and cousin of the unfortunate heroine of "Kenilworth," whose manor of Sidestern descended to Lucy Walpole's son. Can Mr. Francis Galton and the professors of his new science of "Eugenics" derive any lesson from these fragments of genealogy? May we assume that a succession of generations of gentlemen of good estate, trained in the learning and accomplishments of their respective periods, and quietly fulfilling the duties of their several stations, had led to the gradual formation of something like a reservoir of family force, from which, at the critical time, such descendants as Sir Robert Walpole, his brother, his son Horace, and ultimately Nelson, were derived? If the science be worth anything, it ought to throw some light upon such problems as this; and, until it has done so, we may at least take comfort in the possibility of an affirmative reply. The old ballad tells us that "kyng Harry," when he heard of the death of "Persè" at Chevy Chase, said:

> "I have a hondrith captayns in Ynglonde,
> As good as ever was hee."

We have at least no lack of stocks analogous to that from which Nelson sprung, and it is legitimate to hope that some of them at least would, in case of need, furnish a man or men worthy to follow in his steps.

